# Spatial knowledge acquired from first-person and dynamic map perspectives

**DOI:** 10.1007/s00426-020-01389-y

**Published:** 2020-08-09

**Authors:** M. N. A. van der Kuil, A. W. M. Evers, J. M. A. Visser-Meily, I. J. M. van der Ham

**Affiliations:** 1 Department of Health, Medical and Neuropsychology, Leiden University, Wassenaarseweg 52, 2333 AK Leiden, The Netherlands; 2 Center of Excellence in Rehabilitation Medicine, Brain Center, University Medical Center Utrecht, and De Hoogstraat Rehabilitation, Utrecht, The Netherlands; 3 Department of Rehabilitation, Physical Therapy Science & Sports, Brain Center, University Medical Center Utrecht, Utrecht, The Netherlands

## Abstract

As we become familiar with an environment through navigation and map study, spatial information is encoded into a mental representation of space. It is currently unclear to what degree mental representations of space are determined by the perspective in which spatial information is acquired. The overlapping model of spatial knowledge argues that spatial information is encoded into a common spatial representation independent of learning perspective, whereas the partially independent model argues for dissociated spatial representations specific to the learning perspective. The goal of this study was to provide insight into this debate by investigating the cognitive functions underlying the formation of spatial knowledge obtained through different learning perspectives. Hundred participants studied an ecologically valid virtual environment via a first-person and map perspective. The map employed in the study was dynamic, allowing for the disentanglement of learning perspective and sequential information presentation. Spatial knowledge was examined using an array of navigation tasks that assessed both route and survey knowledge. Results show that distinct visuospatial abilities predict route knowledge depending on whether an environment is learned via a first-person or map perspective. Both shared and distinct visuospatial abilities predicted the formation of survey knowledge in the two perspective learning conditions. Additionally, sequential presentation of map information diminishes the perspective dependent performance differences on spatial tasks reported in earlier studies. Overall, the results provide further evidence for the partially dissociated model of spatial knowledge, as the perspective from which an environment is learned influences the spatial representation that is formed.

**Electronic supplementary material:**

The online version of this article (10.1007/s00426-020-01389-y) contains supplementary material, which is available to authorized users.

## Introduction

Whenever we learn about the spatial characteristics of an environment, information is encoded into a mental representation of space (O’Keefe & Nadel, 1978; Tolman, 1948). Research has shown that the nature of a mental representation depends on a variety of factors, such as the navigator’s goal (Brunyé & Taylor, 2009; Taylor, Naylor, & Chechile, 1999), preferred spatial strategy (Pazzaglia & De Beni, 2001), and visuospatial abilities (Hegarty, Montello, Richardson, Ishikawa, & Lovelace, 2006). One factor that is believed to be of particular influence on the characteristics of a mental representation of space is the spatial perspective from which the environment is learned (Richardson, Montello, & Hegarty, 1999; Shelton & Gabrieli, 2002; Thorndyke & Hayes-Roth, 1982; Török, Nguyen, Kolozsvári, Buchanan, & Nadasdy, 2014). The most common method of spatial knowledge acquisition is through direct exploration of an environment. Acquiring spatial information from a first-person perspective tailors to the development of route knowledge (Siegel & White, 1975). Navigators encode spatial information regarding the trajectories between locations in the environment, including sequences of turns, order of landmarks along paths, and landmark–action associations (O’Malley, Innes, & Wiener, 2018). However, we often acquire spatial information from studying indirect sources of information such as cartographic maps. Acquiring spatial information by studying maps directly tailors to the development of survey knowledge of an environment, as cartographic maps depict configurational, layout, and metric information about the relations between landmarks in the environments (Münzer, Zimmer, Schwalm, Baus, & Aslan, 2006).

Although spatial information can be obtained from different perspectives, the emerging mental representations of space go beyond the modality of the learning perspective. Many studies have shown that navigators are able to draw maps of the environment after learning a route from a first-person perspective (Chrastil & Warren, 2012; Coluccia, Bosco, & Brandimonte, 2007; Kozhevnikov, Motes, Rasch, & Blajenkova, 2006; Muffato, Meneghetti, & De Beni, 2020). Additionally, navigators are able to find shortcuts and use place strategies after learning an environment from a first-person perspective, demonstrating configurational knowledge of an environment (Labate, Pazzaglia, & Hegarty, 2014; Wiener, de Condappa, Harris, & Wolbers, 2013). Configurational knowledge of an environment can be obtained even after the initial exposure to an environment (Iglói, Zaoui, Berthoz, & Rondi-Reig, 2009). Conversely, people are able to effectively navigate through an environment and point towards specific locations from a first-person perspective after studying an environment using a map (Allison & Head, 2017; Zhang, Zherdeva, & Ekstrom, 2014).

As such, the consensus is that both route and survey knowledge can be acquired from different learning perspectives. However, there is debate about the cognitive characteristics of mental representations of space acquired via different spatial perspectives (Zhang et al., 2014). One line of evidence suggests that spatial knowledge obtained from different learning perspectives is encoded into a common cognitive representation of space, while the other studies suggests that spatial knowledge obtained from first-person and map perspectives is represented independently. Zhang et al. (2014) classified these different views into a partially independent model and an overlapping model.

The partially independent model is supported by behavioral studies that have shown an advantage for the recall of information congruent with the learning perspective. Spatial knowledge related to routes and trajectories such as route descriptions and route distances are recalled more effective when learned from a first-person perspective compared to a map perspective (Taylor et al., 1999; Thorndyke & Hayes-Roth, 1982). Conversely, map learning leads to higher performance on map sketching and Euclidean distance estimation tasks (Muffato, Meneghetti, & De Beni, 2018; Taylor et al., 1999). As such, spatial representations constructed through first-person navigation are anchored towards the trajectories that have been traversed, whereas representations obtained through maps are more focused towards the configuration of landmarks in the environment (Siegel & White, 1975; Thorndyke & Hayes-Roth, 1982).

The overlapping model proposes that spatial knowledge is encoded into a common representational structure, regardless of the perspective from which an environment is learned. This model is supported by studies that reveal that both first-person and map perspective encoding of spatial information is anchored towards an orientation dependent vector (Shelton & McNamara, 2004).

More recently, the neural mechanisms underlying spatial learning from different perspectives have been studied using neuroimaging techniques. Evidence provided by these studies does not conclusively support one model over the other. Neuroimaging studies report a common neural substrate that is involved in map and first-person perspective learning as well as regions that are distinct for each learning perspective. Some researchers argue that this common neural substrate indicates that spatial information is processed in a mixed or common spatial representation (Latini-Corazzini et al., 2010; Shelton & de Gabrieli, 2002). However, other researchers have focused on the distinct neural substrates, and argue that the existence of different substrates indicates a partially distinct representation of space (Zhang, Copara, & Ekstrom, 2012).

This debate has focused largely on differences between first-person navigation and cartographic map study in terms of perspective modalities. Yet, a fundamental difference between first-person learning and map study is the pacing in which information is presented in both modalities. An inherent property of first-person navigation is that spatial information is presented in a dynamic, sequential fashion. Understanding paths that make up the environment requires navigators to combine and order a set of landmarks and locations. In contrast, during cartographic map study, a complete environment is shown statically. This raises the question whether the differences in mental representations that are observed can be attributed to learning perspectives or to the static and dynamic differences of information presentation. Navigational aids used in cars and mobile devices utilize interactive maps that combine progressive information presentation and map perspectives. These dynamic maps present cartographic information in a route-like fashion and can, thus, serve as a more comparable medium to first-person navigation when studying the effects of perspective learning (Brunyé, Mahoney, & Taylor, 2013).

A few studies have contrasted spatial knowledge acquired through first-person and dynamic map perspectives (Shelton & de Gabrieli, 2002; Shelton & McNamara, 2004; Shelton & Pippitt, 2007; Yamamoto & De Girolamo, 2012). In these studies, spatial representation obtained from different perspective was contrasted after extensive learning of a relatively simple environment. Furthermore, these studies use a limited number of tasks that assess spatial knowledge in each condition.

Insight to this debate might be provided by assessing not only the quality of the spatial knowledge obtained through different learning perspectives, but also by investigating the cognitive mechanisms that underlie spatial learning from different perspectives. Much research has already been directed at determining the contribution of a variety of cognitive functions and visuospatial abilities to first-person and static map study. Most notably, visuospatial working memory, verbal working memory, perspective-taking ability, and mental rotation ability have repeatedly been shown to be involved in navigation (Coluccia et al., 2007; Gras, Gyselinck, Perrussel, Orriols, & Piolino, 2013; Hegarty et al., 2006; Meneghetti, Fiore, Borella, & De Beni, 2011). However, how these functions contribute to spatial knowledge acquisition via different perspectives has yet to be studied systematically. One study contrasted the cognitive mechanisms underlying first-person and dynamic map perspective learning using judgement of relative direction tasks (Fields & Shelton, 2006). This study revealed distinct patterns of visuospatial abilities predicting spatial orientation ability after first-person and dynamic map learning, hinting at a partially dissociated representation. However, to gain more insight into the cognitive mechanism underlying the development of route and survey knowledge from different perspectives, it is important to assess the relationships between visuospatial abilities and a broader array of navigation tasks.

The aim of the current study was to determine to what degree mental representations of space are dependent on learning perspective. We assessed whether overlapping or distinct cognitive functions contribute to performance on a broad range of navigation tasks after first-person and dynamic map learning. To account for the sequential information presentation inherent to first-person navigation, a dynamic map was used to provide spatial information from a map perspective. Spatial knowledge was assessed after a single run through an ecologically valid virtual environment, reflecting a realistic navigation situation in which no overlearning takes place. The previous research provides evidence for both an overlapping and a partially dissociated representation of space after learning from different spatial perspectives. As such, we will examine two hypotheses. If the same set of visuospatial abilities predict performance on the route and survey knowledge tasks regardless of learning perspective, the results support the overlapping model of spatial representation. Conversely, if perspective dependent visuospatial abilities predict performance on route and survey knowledge, we accept the partially independent model. Additionally, we investigated route and survey knowledge obtained through different learning perspectives as the previous studies have interpreted perspective specific advantages as evidence for distinct mental representations of space (Thorndyke & Hayes-Roth, 1982). Following the models of spatial representation, finding a significant advantage of perspective on performance would favor the partially dissociated hypotheses, whereas similar performance would support the overlapping model of spatial representation.

## Methods

### Participants

One hundred participants (63 females) participated in this experiment. Participants were between 18 and 35 years of age (*M* = 22.18, *SD* = 0.28), finished or attended college or university level education. Participants with a history of neurological, psychiatric, and psychological disorders were screened from the experiment (e.g., anxiety disorder, major depression, etc.). All participants signed an informed consent form and were compensated for participation in participant hour credits or with a small monetary reward of 6 euro per hour. The Leiden University’s local ethics committee for psychological research approved this study.

### Materials

The study consisted of two questionnaires, two spatial navigation assessments, and four standardized neuropsychological tests. All computerized components of the study ran on an HP Elite Book 8770 w, with a Core i7-3840QM processor (2.8 GHz) and 16 GB RAM.

### Questionnaires

All participants completed a screening questionnaire in which demographic characteristics such as age, gender, handedness, level of education, and gaming experience was acquired. Furthermore, screening information about a history of psychiatric or neurological disorders was obtained. Subjective navigation complaints were assessed using the Wayfinding Questionnaire (de Rooij, Claessen, van der Ham, Post, & Visser-Meily, 2019). The Wayfinding Questionnaire contains 22 items in 3 subscales: navigation and orientation (11 items), distance estimation (3 items), and spatial anxiety (8 items). All items were rated on a seven-point Likert scale.

### Spatial navigation assessment

**Fig. 1:**
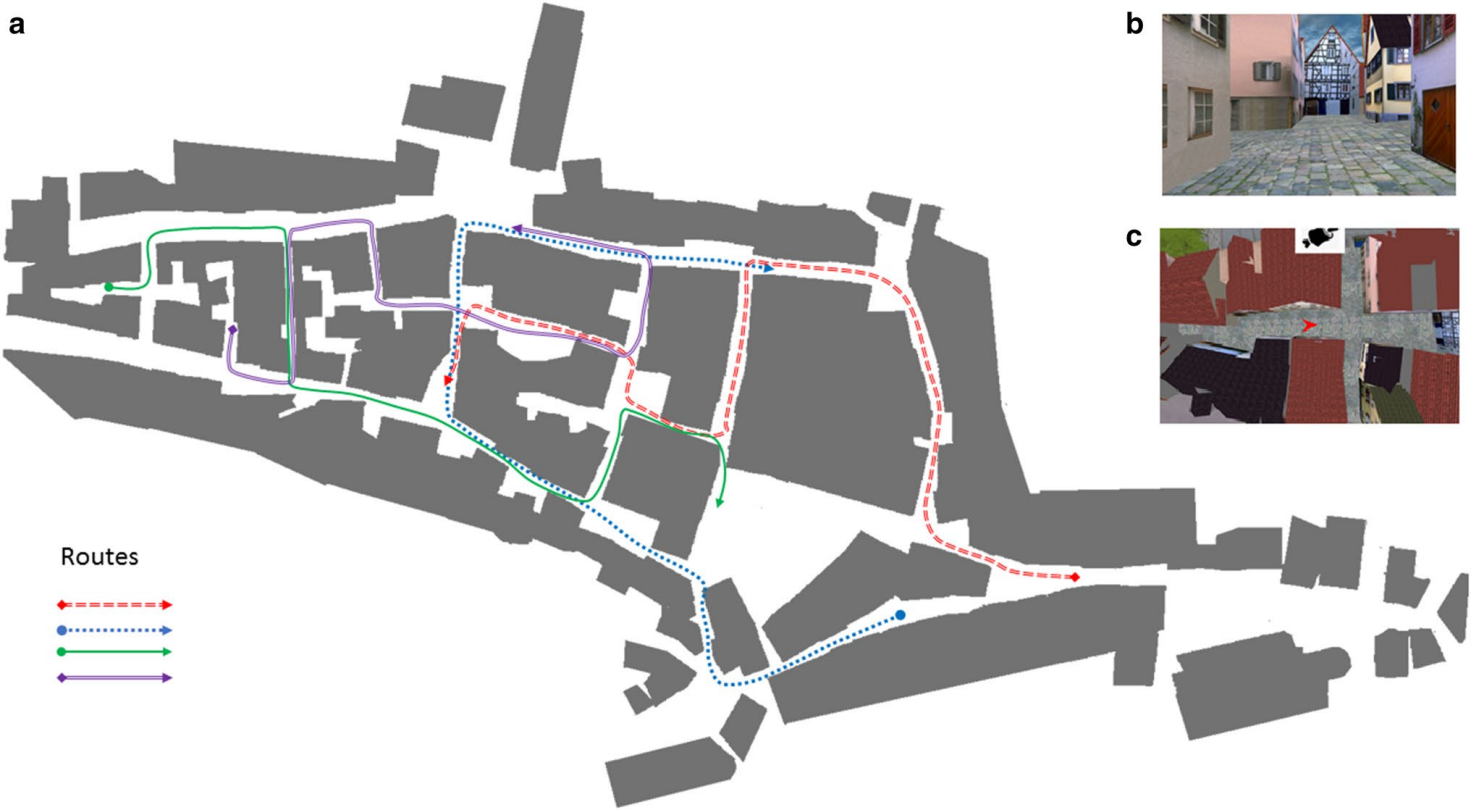
Overview of the environment and the perspectives used in the spatial navigation assessment. **a** Schematic map of the environment. The red, green, blue, and purple lines illustrate the four routes. The arrows indicate the route directions. **b** View of the environment as presented from the first-person perspective. **c** View of the environment as presented from the dynamic map perspective

Objective navigation ability was assessed using an adapted version of the Virtual Tübingen task (Claessen, Visser-Meily, de Rooij, Postma, & van der Ham, 2016). A virtual model of the city center of Tübingen was used as the testing environment (Van Veen, Distler, Braun, & Bülthoff, 1998). Four similar routes through Virtual Tübingen were constructed (Fig. 1a). A comparable distance was traversed in each route (A route: 393 m, B route: 338 m, C route: 371 m, and D route: 367 m). Each route contained eight intersection points. At intersection points, the routes could turn left, right, or go straight ahead. Each route was composed of six unique corridors (paths between intersections) and three common corridors shared with other routes. Common corridors were never visited in the same heading direction in any of the routes.

Each route through the environment could be shown from two perspectives: first-person perspective (Fig. 1b) and dynamic map perspective (Fig. 1c). In the first-person perspective variation, participants observed the route from a camera placed at a height of 1.70 m. At each intersection, the camera would stop and turn in the direction of each corridor before continuing along the route. In the dynamic map perspective variation, a red arrow was shown on the map that traversed the environment. This arrow was shown from an aerial, bird’s-eye view (38 m high), using a camera locked onto the position of the arrow. The camera was always aligned towards the north and did not rotate. An orthographic lens was used, revealing the walls of the buildings of corridors in the environment. Eight black and white icons were placed above buildings to indicate a buildings’ function (e.g., theatre, library, etc.). During the learning phase, participants were instructed to memorize as much as possible about the spatial characteristics of the environment.

### Navigation tasks

After learning the environment (from either first-person or dynamic map perspective), participants completed six recall tasks in which navigation abilities were assessed. The first two tasks, *Route Sequence* and *Route Continuation*, assessed route knowledge. The remaining four tasks, *Point to Start location*, *Point to End location*, *Distance Comparison,* and *Locations on Map,* measured survey knowledge.

Directly after observing the video, a Route Sequence task was conducted. Participants indicated what action was taken at each of the eight intersections. Options were left-turn, right-turn, or straight ahead. No images of the intersections were shown. Numbers 1–8 were listed and participants selected the arrow icon indicating the response options. This task required an egocentric reference frame as a number of bodily turns were requested regarding the navigator in the environment. In the map-perspective condition, an orientation switch was required, as the turn direction was based on the orientation of the red arrow that moved along the route. A participant’s score was the sum of correct responses (ranging from 1 to 8).

Then, the Route Continuation task was performed. Participants were presented with eight images of the intersections in random order. Participants had to indicate whether they turned left, right, or went straight ahead at each decision point by pressing the arrow keys left, right, or up, respectively. In the map-perspective condition, an orientation switch was required, as the turn direction was based on the orientation of the red arrow that moved along the route. A participant’s score was the sum of correct responses (ranging from 1 to 8).

Participants then performed the Point to Start and Point to End tasks. Participants were shown eight scenes taken along the route in random order. Participants were asked to indicate where the start or end locations of the route were using a rotational device. In the first-person perspective variation, the rotational device was placed horizontally on the desk in front of the participants. Participants were asked to point from the perspective shown in the image. In the map perspective version, the rotational device was placed vertically on the desk next to the monitor. Participants had to indicate the start/ending location on the map, relative to the red arrow icon the camera was following. The perspective from which the items of the task were presented corresponded to the perspective in which the environment was learned (no perspective switch was enforced). As such, the spatial orientation tasks assess survey knowledge in both learning perspectives (Ekstrom, Arnold, & Iaria, 2014). Scoring was based on the mean pointing deviation angle for each trial, ranging from 0 to 180 degrees deviation.

In the Distance Comparison task, participants completed eight trials in random order. In each trial, a target image and two response images were shown. In the first-person perspective version, the images were scenes along the route. In the map perspective version, the images were landmarks encountered along the route. Participants had to indicate which of the two response locations was closest to the target location (crow’s flight distance). This task required an allocentric reference frame to complete as metric, configurational knowledge of the environment was assessed. Scoring was based on the number of correct responses (ranging from 1 to 8).

The final task participants performed was Locations on Map. Participants were shown a schematic city map including icons indicating starting and ending locations. In the first-person perspective version, participants were shown images of eight scenes along the route in random order. Participants had to indicate the correct location on the city map using the mouse. In the map perspective version, participants had to indicate where landmarks were located on the city map. Scoring was based on the mean distance deviation from the correct location (pixels) for each trial.

### Neuropsychological assessment

Four neuropsychological tests were performed to assess visuospatial abilities. The forward and backwards Corsi block-Tapping tasks were used to assess visuospatial working memory (Kessels, van den Berg, Ruis, & Brands, 2008; Kessels, van Zandvoort, Postma, Kappelle, & de Haan, 2000). Product score (span x item score) was calculated and used as outcome measure.

The WAIS-IV Digit Span test was used to assess verbal attention span and working memory (Wechsler, 2008). Product score (span × item score) was calculated and used as outcome measure.

A computerized version of the Mental Rotation task was used assess higher level visuospatial processing (Shepard & Metzler, 1971). The version of the mental rotation tasks contained 48 trials. Half of the trials contained pairs of images that depicted the same object, whereas the other trials contained mirrored pairs. The rotation used for the objects were 0, 45, 90, and 180 degrees mental rotation over either the horizontal or vertical axes. Total correct answers (accuracy) were taken as outcome variable. Reaction time slope and intercept for correct answers were calculated using the “least squares” method to calculate a straight line over the reaction times for the different degrees of mental rotation. Note that eight participants did not have a correct answer on all of the stimuli categories. For these cases, a line was fitted on the available data.

The Santa Barbara Object Perspective-Taking Tests were used to measure perspective-taking ability. The task contained 12 items. Average pointing deviation in degrees was calculated as main outcome variable for this task (Hegarty & Waller, 2004).

### Procedure

All participants read the study’s information letter and signed an informed consent form prior to the experimental session in concordance with the Declaration of Helsinki (2013). The session started by filling in the screening questionnaire, followed by the Wayfinding Questionnaire. Participants then completed the navigation tasks. Two of the four available routes were assigned to each participant in a counter balanced procedure based on enrolment order (eight possible combinations of routes). For each route, a map perspective and a first-person perspective version was available. Participants would observe the two routes from different learning perspectives. Half of the participants would start with the dynamic map perspective, while the other half started with the first-person perspective. Participants observed the demo route and completed the subtests in the following order: Route Sequence, Route Continuation, Distance Comparison, Point to Start, Point to End, and Location on Map. This order of tasks was maintained to minimize the transfer of knowledge obtained through questions (e.g., the Route Continuation task contained information beneficial for the Route Sequence task). After the first demo video and tasks were completed, the procedure with the alternative perspective was performed. A 15-min break was introduced after which participants completed the visuospatial tests in the following order: Corsi Block Tapping, Digit Span, Santa Barbara perspective taking, and the Mental Rotation task.

### Statistical analysis

The mean scores on the neuropsychological tests (Corsi Block Tapping, Digit Span, Mental Rotation, and Perspective Taking) and the navigation tasks (Route Sequence, Route Continuation, Point to Start, Point to End, Distance Comparison, and Locations on Map) were calculated. Then, the relationship between neuropsychological abilities and performance on navigational tasks was investigated for both perspectives. First, a Pearson correlation analyses was performed to explore the relationship between all variables. This was followed by exploratory backward stepwise linear regression analyses that included (1) gender, (2) perspective-taking task score, the product score of (3) forward and (4) backward Corsi Block Tapping task, the product score of (5) forward and (6) backward Digit Span product scores, (7) Mental Rotation accuracy, (8) slope, and (9) reaction time, as independent variables. Performance on the 12 navigation tasks was used as dependent variables. The elimination criteria for these regression models were set to *p *< 0.1. All assumptions of multiple regression were assessed and met.

Performance differences on navigation tasks between different learning perspectives were assessed using a mixed model MANCOVA analysis, with learning perspective (first-person vs. dynamic map) as within-subject factor. Gender was included as a between subject factor. The scores of the neuropsychological tests were included as covariates.

Responses in the navigation tasks with a reaction time faster than 200 ms were negated. Average scores for each task were calculated without these trials. This occurred in 10/4800 trials (0.2%). Due to technical difficulties, the data of two participants were missing for the Route Sequence task (dynamic map perspective). To minimize the effects of extreme values in the regression analyses, Point to Start and Point to End (dynamic map perspective) were transformed using a 10 log transformation. Point to Start and Point to End (first-person perspective) were transformed using a square-root transformation.

## Results

Demographic data and an overview of neuropsychological test performance are presented in Table 1. Overall performance scores on the navigation subtasks for both the first-person and dynamic map perspectives are displayed in Table 2. The results of the exploratory Pearson correlation analysis are presented in Supplementary Table 1. Diagnostics of the multiple regression assumption tests are displayed in Supplementary Tables 2 and 3.

**Table 1:** Demographics and scores on neuropsychological tests

Variable	*M*	*SD*
Demographic		
Age	22.18	2.81
Gender (% male)	37	
Education*	6.62	0.49
Neuropsycholigcal assessment		
Perspective-taking test (deviation)	24.10	14.91
Corsi span, forward (span)	6.43	0.95
Corsi span, forward (product)	64.22	19.44
Corsi span, backward (span)	6.57	0.83
Corsi span, backward (product)	67.63	17.58
Digit span, forward (span)	6.41	1.33
Digit span, forward (product)	64.93	26.96
Digit span, backward (span)	5.37	1.13
Digit span, backward (product)	52.70	21.24
Mental rotation, accuracy (%)	76.73	11.51
Mental rotation, reaction time (ms)	5309.24	3591.95
Mental rotation, slope (ms/degree)	20.83	15.23

*Education measured using the Verhage scale, a classification of education according to the Dutch education system. Ranging from 1–7, with 7 being the highest education level

**Table 2:** Performance scores on navigation subtasks for both the dynamic map perspective and first-person perspective conditions

Virtual Tübingen tasks	Dynamic map perspective		First-person perspective		MANCOVA			Post hoc contrast
	*M*	*SD*	*M*	*SD*	*DF*	*F*	*p*	*p*
Route knowledge					2, 87	0.102	0.903	
Route sequence (% correct)	65.69	24.57	61.13	28.81				–
Route continuation (% correct)	82.14	16.76	69.75	19.48				–
Survey knowledge					2, 89	0.089	0.915	
Distance comparison (% correct)	66.75	17.15	65.13	20.59				–
Location on map (deviation in pixels)	120.98	69.09	142.72	73.52				–
Survey knowledge (orientation tasks)					*	*	*	
Point to start location (deviation in degrees)	24.58	25.21	49.29	20.56				< 0.001
Point to end location (deviation in degrees)	28.18	15.24	51.78	23.12				< 0.001

*Data did not meet assumptions for MANCOA analysis; post hoc contrast calculated using a signed rank *t* test

### Regression and performance analysis

#### Route knowledge tasks

Multiple regressions were calculated to determine whether similar of different small-scale spatial abilities predicted performance on the *Route Sequence* and *Route Continuation* tasks after learning a route from a dynamic map and first-person perspective (Table 3).

**Table 3:** Results of stepwise multiple regression analyses of factors predicting performance on route knowledge tasks after learning a route from first-person and dynamic map perspectives

Nav. task	Predictors	*t*	*p*	*B* (*SE*)	*β*	*F*	*p*	*R*^*2*^
First-person perspective								
Route Sequence	Overall model					7.75	**< 0.01**	0.14
	Corsi forward (product)	3.63	< **0.01**	0.51 (0.14)	0.34			
	Mental rotation (slope)	1.89	< 0.1	0.34 (0.18)	0.18			
Route continuation	Overall model					7.28	**< 0.01**	0.13
	Perspective taking	− 1.76	< 0.1	− 0.22 (0.13)	− 0.17			
	Corsi forward (product)	3.05	< **0.01**	0.29 (0.09)	0.29			
Dynamic map perspective								
Route Sequence	Overall model					5.25	**< 0.01**	0.10
	Perspective taking	− 2.39	< **0.05**	− 0.39 (0.16)	− 0.24			
	Corsi forward (product)	1.81	< 0.1	0.23 (0.13)	0.18			
Route continuation	Overall model					10.81	**< 0.01**	0.18
	Perspective taking	− 3.63	< **0.01**	− 0.39 (0.11)	− 0.35			
	Mental rotation (accuracy)	1.84	< 0.1	0.26 (0.14)	0.18			

Significant *p*-values printed in bold

##### Route Sequence

A significant model was found for Route Sequence (first-person perspective), *F* (2, 97) = 7.75, *p* < 0.001, with a *R*^2^ of 0.14. Corsi forward product score significantly predicted Route Sequence score in the first-person learning condition (*p *< 0.001). Participant’s Route Sequence score increased score by 0.51 for each increment of Corsi forward product score. As the variable elimination criteria were set to *p* < 0.1, the model included a trend-level interaction between Mental Rotation (slope) and Route Sequence score (*p *= 0.061).

A significant regression equation was found for Route Sequence (dynamic map perspective), *F* (2, 95) = 5.25, *p *< 0.01 with a *R*^2^ of 0.10. Perspective-taking score significantly predicted Route Sequence score in the map learning condition (*p *< 0.05). Participant’s Route Sequence score decreased by 0.39 for each degree of deviation in the perspective-taking task. None of the other variables significantly predicted Route Sequence score. The model included a trend-level interaction between Corsi forward product score and Route Sequence score (*p *= 0.073).

##### Route continuation

A significant regression model was found for Route Continuation (first-person perspective), *F* (2, 97) = 7.28, *p *< 0.01 with a *R*^2^ of 0.13. Corsi forward product significantly predicted Route Continuation score in the first-person learning condition (*p *< 0.01). Participant’s Route Continuation score increased by 0.29 for each increment of Corsi forward product score. None of the other variables significantly predicted Route Continuation (first-person learning). The model included a trend-level interaction between Perspective taking and Route Continuation score (*p* = 0.081).

A significant regression model was found for Route Continuation (dynamic map perspective), *F* (2, 97) = 10.81, *p *< 0.001 with a *R*^2^ of 0.18. Perspective-taking score significantly predicted Route Sequence score in the map perspective condition (*p *< 0.001). Participant’s Route Continuation score decreased by 0.39 for each degree of deviation in the perspective-taking task. None of the other variables significantly predicted Route Continuation (dynamic map perspective). The model included a trend-level interaction between Mental rotation accuracy and Route Continuation score (*p* = 0.069).

A mixed model MANCOVA was performed to assess the effect of learning perspective on performance of the route knowledge tasks. The MANCOVA did not reveal a main effect for learning perspective on performance in the tasks (*p *> 0.05) (Table 2). A significant interaction effect was found for learning perspective * Corsi Forward Product, *F* (2, 87) = 5.95, *p* < 0.01 partial *η*^2^ = 0.12. Univariate tests showed a significant interaction effect of perspective and Corsi Forward product score on the Route Continuation performance, *F* (1, 88) = 10.13, *p* < 0.01 partial *η*^2^= 0.10.

#### Survey knowledge tasks

Multiple regressions were calculated to determine whether similar of different small-scale spatial abilities predicted performance on the *Distance Estimation*, *Location on Map, Point to Start,* and *Point to End* tasks after learning a route from a dynamic map and first-person perspective (Table 4).

**Table 4:** Results of stepwise multiple regression analyses of factors predicting performance on survey knowledge tasks after learning a route from first-person and dynamic map perspectives

Nav. task	Predictors	*t*	*p*	*B* (*SE*)	*β*	*F*	*p*	*R*^2^
First-person perspective								
Distance estimation	Overall model					–	n.s.	–
Location on map*	Overall model					6.32	**< 0.01**	0.17
	Gender	1.94	< 0.1	27.56 (14.22)	0.18			
	Corsi forward (product)	− 3.78	< **0.01**	− 1.35 (0.36)	− 0.36			
	Mental rotation (slope)	− 1.67	< 0.1	− 0.76 (0.45)	− 1.57			
Point to start^†^	Overall model					7.50	**< 0.001**	0.24
	Gender	2.67	< **0.01**	0.74 (0.28)	0.25			
	Perspective taking	2.69	< **0.01**	0.03 (0.00)	0.25			
	Corsi forward (Product)	− 2.97	< **0.01**	− 0.02 (0.00)	− 0.27			
	Mental rotation (RT)	− 1.67	< 0.1	− 0.0(0.00)	− 0.15			
Point to end^†^	Overall model					8.93	**< 0.001**	0.22
	Perspective taking	1.73	< 0.1	0.02 (0.01)	0.16			
	Corsi forward product	− 3.46	< **0.01**	− 0.03 (0.00)	− 0.32			
	Mental rotation (accuracy)	− 1.85	< 0.1	− 0.03 (0.01)	− 0.18			
Dynamic map perspective								
Distance estimation	Overall model					3.83	< 0.1	0.03
	Corsi backward (product)	1.96	< 0.1	0.19 (0.09)	0.19			
Location on map*	Overall model					4.60	**< 0.05**	0.05
	Corsi Backward (product)	− 2.15	< **0.05**	− 0.83 (0.39)	− 0.21			
Point to start^‡^	Overall model					30.69	**< 0.001**	0.24
	Perspective taking	5.54	< **0.01**	0.01 (0.002)	0.48			
Point to end^‡^	Overall model					12.31	**< 0.001**	0.20
	Perspective taking	4.01	< **0.01**	0.005 (0.00)	0.37			
	Corsi forward product	− 2.23	< **0.05**	− 0.002 (0.00)	− 0.21			

Significant *p* values printed in bold

*n.s*. indicates non-significant, non-trend

*Outcome measured in deviation. A lower score indicated a higher performance

^†^Data were square root transformed

^‡^Data were log transformed

##### Distance comparison

No significant predictors were found for performance on the Distance Comparison task in the first-person learning perspective. The backward elimination procedure removed all independent variables with a *p* score larger than 0.10. Similarly, no significant predictors of Distance Comparison were found after learning from a dynamic map perspective. A model with trend-level significance resulted from the backwards elimination procedure *F* (1, 98) = 3.83, *p *= 0.053 with a *R*^2^ of 0.03. The Corsi Backward product score predicted Distance Estimation at trend level (*p* = 0.053).

##### Location on map

A significant regression model was found for the Location on Map task (first-person learning), *F* (3, 96) = 6.32, *p *< 0.01 with a *R*^2^ of 0.17. Corsi forward product significantly predicted Location on Map score in the first-person learning condition (*p *< 0.01). Participant’s Location on Map accuracy (measured in pixel deviation) increased by 1.35 for each increment of Corsi forward product score. None of the other variables significantly predicted Location on Map (first-person learning). The model included two trend-level relations with Location on Map score: Gender (*p *= 0.056) and Mental Rotation (slope) (*p* = 0.09).

A significant regression model was found for the *Location on Map* task (dynamic map perspective), *F* (1, 98) = 4.60, *p *< 0.05 with a *R*^2^ of 0.05. Corsi Backward product score significantly predicted *Location on Map* score in the map perspective condition (*p *< 0.05). Participant’s Location on Map accuracy (measured in pixel deviation) increased by 0.83 for each increment of Corsi backward product score. The regression models reveal that visuospatial working memory, as measured in the Corsi Forward and Backward block-Tapping task, predicted performance in survey tasks after learning from both the first-person and dynamic map perspectives.

A mixed model MANCOVA was performed to assess the effect of learning perspective on performance on Distance Estimation and Location on Map. The MANCOVA did not reveal a main effect for learning perspective on performance in both tasks (*p *> 0.05) (Table 2).

##### Pointing to start location

A significant regression model was found for Point to Start (first-person perspective), *F* (3, 95) = 7.5, *p *< 0.001 with a *R*^2^ of 0.24. Perspective taking (*p *< 0.01), Gender (*p *< 0.01), and Corsi Forward product score (*p *< 0.01) significantly predicted Point to Start score after first-person learning. Square-root transformed pointing deviation increased by 0.03 degrees for each degree of pointing deviation in the perspective-taking task. Square-root transformed Pointing deviation decreased by 0.02 degrees for each increment of Corsi forward product score.

A significant regression model was found for Point to Start (dynamic map perspective), *F* (1, 98) = 30.69, *p *< 0.001 with a *R*^2^ of 0.24. Perspective taking significantly predicted Point to Start score in the map perspective condition (*p *< 0.001). Log transformed pointing deviation increased by 0.01 degrees for each degree of pointing deviation in the perspective-taking task. None of the other variables significantly predicted Point to Start.

##### Pointing to end location

A significant regression model was found for Point to End (first-person perspective), *F* (3, 96) = 8.93, *p *< 0.01 with a *R*^2^ of 0.22. Corsi forward product significantly predicted Point to End score in the first-person learning condition (*p *< 0.001). Square-root transformed pointing deviation decreased by 0.03 degrees for each increment of Corsi forward product score. None of the other variables significantly predicted Point to End. The model included two trend-level relations for Perspective Taking (*p *= 0.087) and Mental Rotation (accuracy) (*p *=* 0*.067).

A significant regression model was found for Point to End (dynamic map perspective), F (2, 97) = 12.31, *p *< 0.01 with a *R*^2^ of 0.2. Corsi forward product significantly predicted Point to End score in the map perspective condition (*p *< 0.05). Log transformed pointing deviation increased by 0.002 degrees for each increment of Corsi forward product score. Additionally, perspective taking significantly predicted Point to End score in the map perspective condition (*p *< 0.001). Log transformed pointing deviation increased by 0.005 degrees for each degree of pointing deviation in the perspective-taking task.

Due to a non-normal distribution of the Point to Start and Point to End data (in both perspective groups), the assumptions of a mixed model MANCOVA were not met. Therefore, Wilcoxon signed rank tests were conducted assess the effect of learning perspective on performance on the orientation tasks (Table 2). The Wilcoxon signed rank tests revealed a significant effect of perspective on performance on both the Point to Start (*Z* = − 7.65, *p* < 0.001) and Point to End tasks (*Z* = − 7.32, *p* < 0.001). Performance was significantly higher in the dynamic map perspective condition compared to the first-person perspective condition in both Point to Start (*M* = 24.58, *SD* = 25.21 vs. *M* = 49.29, *SD* = 20.56) and Point to End tasks (*M* = 28.18, *SD* = 15.24 v*s. M* = 51.78, *SD* = 23.12).

**Fig. 2:**
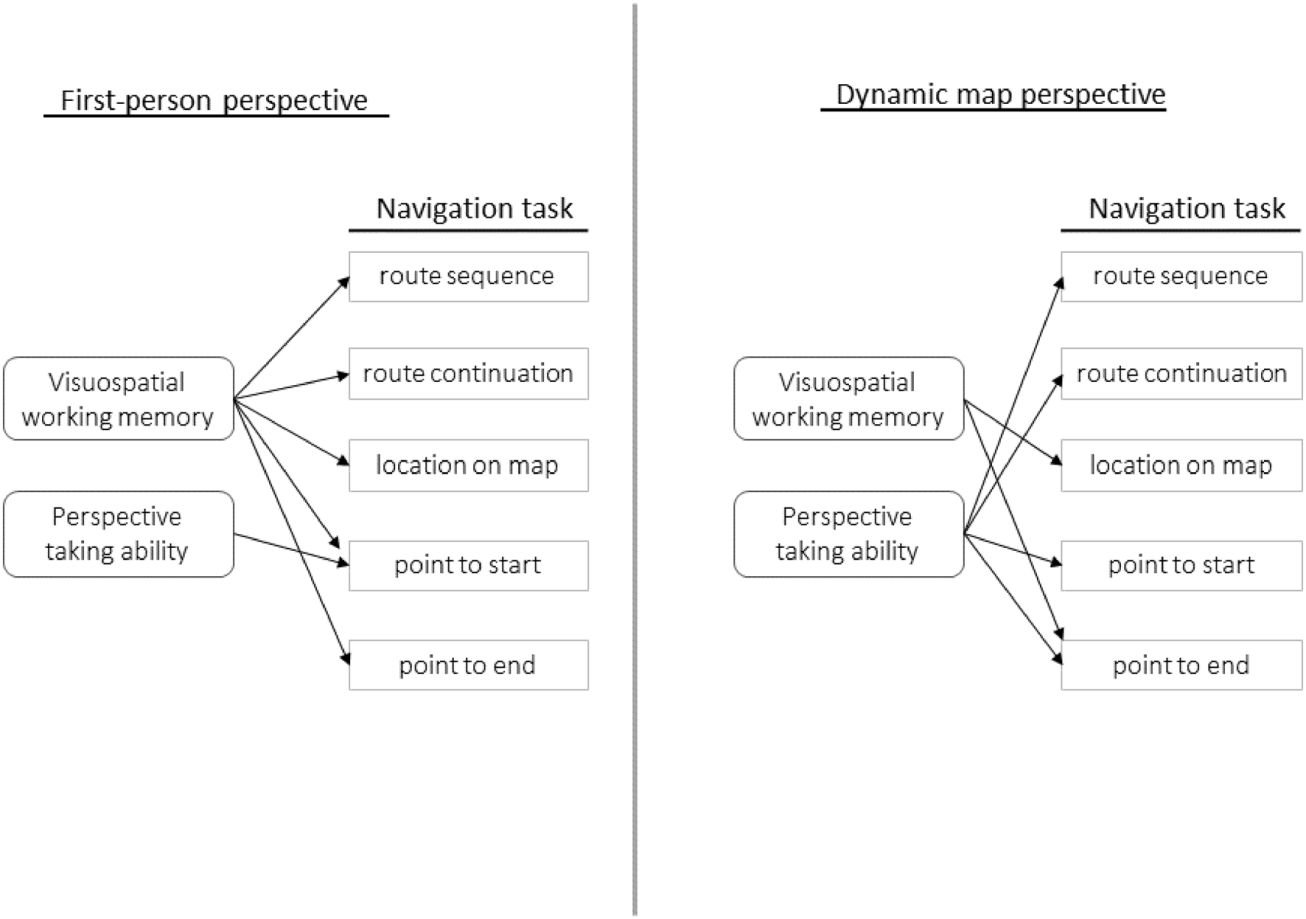
Summary of visuospatial task predicting performance on navigation tasks per perspective as obtained in regression models. Arrows indicate a significant predictive relationship of the visuospatial task on the navigation task

A schematic overview of the visuospatial tasks predicting performance on navigation subtasks is presented in Fig. 2. Overall, the results reveal distinct patterns of visuospatial abilities predicting performance on route knowledge tasks for the two perspectives. Conversely, both shared and distinct visuospatial abilities predict performance on survey knowledge tasks in the two learning perspectives.

## Discussion

Within the field of spatial cognition, there is debate revolving around the influence of learning perspective on the characteristics of mental representations of space (Zhang et al., 2014). One line of research suggests that spatial information is stored in a common representation of space (Latini-Corazzini et al., 2010; Shelton & McNamara, 2004), while other evidence points towards partially dissociable representations of space that are dependent on learning perspective (Taylor et al., 1999; Thorndyke & Hayes-Roth, 1982). The aim of the current study was to determine whether a common representation of space was formed when information was learned via different perspectives by assessing relations between visuospatial abilities and different types of spatial knowledge. The overlapping model of spatial representation would predict that the same visuospatial abilities would predict performance on the route and survey knowledge tasks after first-person and dynamic map learning. Conversely, the (partially) independent model would predict that different visuospatial abilities would predict performance on the route and survey knowledge tasks.

Our results indicate that distinct visuospatial abilities underlie the formation of route knowledge after learning from a first-person and dynamic map perspective. Visuospatial working memory predicted performance on the route knowledge tasks in the first-person learning condition, whereas perspective-taking ability predicted performance on route knowledge tasks in the dynamic map perspective condition. The importance of visuospatial working memory in the formation of route knowledge during direct navigation has been observed in the other studies (Garden, Cornoldi, & Logie, 2002; Meneghetti et al., 2016; Wen, Ishikawa, & Sato, 2011). It has been suggested that visuospatial working memory is responsible for storing and processing of spatial information and facilitating other visuospatial abilities (Meneghetti et al., 2016). perspective-taking ability is believed to play an important role in acquiring knowledge about locations and readjustment of orientation during information processing (Hegarty et al., 2006). This ability is predominantly involved in processing of configurational representation of spatial information. However, the ability has been shown to contribute to route knowledge (Kozhevnikov et al., 2006). The distinction in cognitive processes contributing to performance on the route knowledge tasks shows that route knowledge is processed differently depending on learning perspective. The involvement of visuospatial working memory in the first-person learning conditions suggests that participants recall the sequence of events in the video without computing perspective changes or transformations. The involvement of spatial transformation in the map perspective condition, suggest that participants adjusted their orientation on the mental image of the environment to complete the tasks (Fields & Shelton, 2006; Meneghetti et al., 2011). Therefore, it seems likely that route knowledge obtained through the first-person navigation is stored into an egocentric reference frame, whereas route knowledge obtained through dynamic map perspective is stored into a more allocentric oriented reference frame.

Assessment of the visuospatial abilities underlying performance on the survey knowledge tasks reveals a more complex interaction. While there are shared visuospatial abilities predicting survey knowledge in the first-person and dynamic map perspectives, there are also relations that are specific to the perspective conditions. In both perspective conditions, visuospatial working memory contributed to performance on configurational knowledge of the environment, in accordance with studies that studied the role of visuospatial working memory in static map study and direct navigation designs (Coluccia et al., 2007; Garden et al., 2002; Muffato et al., 2020; Wen et al., 2011). Furthermore, perspective-taking ability predicted performance on the orientation tasks in both perspective learning conditions. These results closely resemble an earlier study in which cognitive mechanisms underlying first-person and dynamic map perspective learning using a judgement of relative direction tasks were studied (Fields & Shelton, 2006). There are, however, distinct predictors of spatial knowledge related to the learning perspective. Visuospatial working memory predicted ‘pointing to start’ ability after first-person learning, which was not observed after dynamic map learning. Conversely, visuospatial working memory predicting ‘point to end’ performance in the dynamic map learning, which was not observed after first-person learning. As such, we argue that there are both overlapping and distinct cognitive processes in both learning perspectives. This suggests that survey knowledge acquired through first-person information is encoded and processed using a mental representation that is *at least* partially distinct from information obtained through map learning.

Perspective-dependent advantages of on route and survey tasks have been taken as evidence for differential spatial representations (Zhang et al., 2014). These studies have contrasted performances after static map study with direct navigation. However, when introducing sequential pacing of information in the map learning perspective, the quality of route knowledge is comparable to the first-person navigation. In contrast to the previous studies that employed cartographic maps and first-person learning (Taylor et al., 1999; Thorndyke & Hayes-Roth, 1982), no advantage for first-person perspective learning over map perspective learning was found on performance on route knowledge tasks. These results are in line with more recent studies that employed a similar method of map presentation and route knowledge assessment (Muffato et al., 2020). Following Muffato et al. (2020), we argue that these discrepancies arise as a result of the presentation of a route information in the map perspective condition, as compared to a static cartographic map.

Mixed results were found for the advantages of learning perspectives on tasks that assessed survey knowledge of the environment. No performance differences were found for survey knowledge tasks that assessed the locations of landmarks in the environment, whereas an advantage for the dynamic map perspective was found on the orientation tasks that assessed relative directions between locations. Perspective-dependent advantages for configurational knowledge have been demonstrated in many studies (Muffato et al., 2020; Shelton & Pippitt, 2007; Taylor et al., 1999; Thorndyke & Hayes-Roth, 1982; Yamamoto & De Girolamo, 2012). In line with these studies, our results support the notion that survey knowledge is processed differently depending on learning perspective. The results should be interpreted with caution, however. The stimuli used in the current tasks always corresponded to the perspective in which the environment was learned to minimize the prompting of a representation other than the input perspective (Muffato et al., 2020). This, however, required participants in the first-person perspective to take the heading direction into account when pointing to different locations, which was not the case in the dynamic map perspective. The additional cost of switching between the first-person stimulus presentation (egocentric) in the task and the allocentric representation in which information was encoded might explain the performance differences between learning perspectives (Lee & Tversky, 2001).

Overall, this study provides further evidence for the model that states that mental representations of space are dependent on learning perspective. While this result contributes to the theoretical understanding of navigation ability, it has implications for more applied research. There have been attempts to develop diagnosis tools and treatments for neurological patients (i.e., acquired brain injury, Alzheimer’s disease) with navigation impairments (Bouwmeester, van de Wege, Haaxma, & Snoek, 2015; Cogné et al., 2017; Kober et al., 2013). Our results stress the importance of using a comprehensive set of navigation tests in the diagnosis of these impairments. Route knowledge, in particular, should be assessed using both first-person and map-based perspectives. In terms of treatment selection, our results support the idea of a compensationary approach to navigation impairments (Claessen, van der Ham, Jagersma, & Visser-Meily, 2016). The dissociable nature of route knowledge suggests that route knowledge impaired patients might benefit from using maps. Conversely, participants with survey knowledge impairments can be trained to develop a navigation strategy focusing on the acquisition of route knowledge from a first-person perspective.

The current study contained some limitations that must be mentioned. First, the Virtual Tübingen environment was limited in spatial dimensions. To keep the properties (length and number of intersections) of the routes similar, it was inevitable that parts of the routes overlapped. This overlap was kept to a minimum and kept similar between routes: all routes contained one overlapping street (path between two intersections) with one other route. This overlapping street was never visited in the same travelling direction. Regardless, it is possible that spatial information leaked over between the two perspective learning conditions. If this was the case, the task would be slightly biased towards the hypothesis supporting the common representation of space. However, as the results support the partial dissociation hypothesis, we argue that the overlap in routes had a minimal impact on the results. Second, the current study provides a comprehensive comparison of visuospatial and cognitive functions underlying a broad array of spatial abilities under different learning conditions. While the current selection of visuospatial and spatial abilities tasks cover the main components of spatial navigation, the assessment is far from complete. To gain a more complete understanding of the mental representations of space and how these are constructed under different learning perspectives, future studies should investigate the mechanisms underlying map sketching, scene/landmark recognition, and route completion abilities. Finally, to maximize the similarities between perspective learning conditions, the current study was limited to passive learning of the environment. Active navigation allows participants to learn an environment in a more ecologically valid manner as participants can utilize preferred and familiar spatial strategies (Chrastil & Warren, 2012). This approach might provide a more detailed insight into the cognitive mechanisms underlying the construction of mental representations.

When placing our findings in context of the two main models on the nature of spatial information, the overlapping model and partially dissociation model, we are able to contribute a novel observation. Distinct cognitive functions underlie route knowledge when information is obtained through first-person or map learning perspectives. Partially distinct cognitive functions underlie survey knowledge in the two perspective learning condition. Additionally, when including a sequential pacing of information in map perspective learning and using a sufficiently complex environment, the observed advantages for first-person learning on route knowledge acquisition and map learning for survey knowledge diminish. Overall, our results support the notion that both route and survey knowledge representations are dissociated for different learning perspectives.

## Electronic supplementary material

Below is the link to the electronic supplementary material.

Supplementary material 1 (DOCX 21 kb)

Supplementary material 2 (PDF 354 kb)

Supplementary material 3 (PDF 354 kb)
