# Supplementary material for: Spatial knowledge acquired from first-person and dynamic map perspectives
Source: Psychol Res. 2020 Aug 9;85(6):2137–50. doi: 10.1007/s00426-020-01389-y (PMC8357693; doi:10.1007/s00426-020-01389-y)
Supplement: Supplementary file 1 — Supplementary material 1 (DOCX 21 kb) [file 426_2020_1389_MOESM1_ESM.docx]

Supplementary Table 1. Pearson correlations matrix for navigation subtasks and neuropsychological tests.

| Variable | 1 | 2 | 3 | 4 | 5 | 6 | 7 | 8 | 9 | 10 | 11 | 12 | 13 | 14 | 15 | 16 | 17 | 18 | 19 | 20 |
| --- | --- | --- | --- | --- | --- | --- | --- | --- | --- | --- | --- | --- | --- | --- | --- | --- | --- | --- | --- | --- |
| 1. Route Sequence (DMP) | 1 |  |  |  |  |  |  |  |  |  |  |  |  |  |  |  |  |  |  |  |
| 2. Route Sequence (FPP) | 0.11 | 1 |  |  |  |  |  |  |  |  |  |  |  |  |  |  |  |  |  |  |
| 3. Route Continuation (DMP) | 0.05 | 0.09 | 1 |  |  |  |  |  |  |  |  |  |  |  |  |  |  |  |  |  |
| 4. Route Continuation (FPP) | 0.19 | **0.21^*^** | **0.38^**^** | 1 |  |  |  |  |  |  |  |  |  |  |  |  |  |  |  |  |
| 5. Distance Comparison (DMP) | 0.14 | **0.25^*^** | 0.07 | 0.13 | 1 |  |  |  |  |  |  |  |  |  |  |  |  |  |  |  |
| 6. Distance Comparison (FPP) | -0.03 | 0.19 | **0.35^**^** | **0.28^**^** | 0.13 | 1 |  |  |  |  |  |  |  |  |  |  |  |  |  |  |
| 7. Location on Map (DMP) | **-0.22^*^** | -0.15 | -0.19 | **-0.32^**^** | **-0.34^**^** | **-0.27^**^** | 1 |  |  |  |  |  |  |  |  |  |  |  |  |  |
| 8. Location on Map (FPP) | 0.04 | **-0.25^*^** | -0.19 | **-0.49^**^** | **-0.26^**^** | **-0.46^**^** | **0.24^*^** | 1 |  |  |  |  |  |  |  |  |  |  |  |  |
| 9. Point to Start (DMP) † | **-0.28^**^** | -0.14 | **-0.35^**^** | **-0.21^*^** | -0.08 | **-0.26^**^** | **0.22^*^** | **0.24^*^** | 1 |  |  |  |  |  |  |  |  |  |  |  |
| 10. Point to Start (FPP) ‡ | -0.08 | **-0.26^**^** | -0.15 | **-0.31^**^** | -0.11 | **-0.22^*^** | 0.18 | **0.41^**^** | **0.3^**^** | 1 |  |  |  |  |  |  |  |  |  |  |
| 11. Point to End (DMP) † | **-0.32^**^** | **-0.25^*^** | **-0.26^**^** | **-0.28^**^** | -0.13 | **-0.29^**^** | **0.47^**^** | **0.25^*^** | **0.46^**^** | **0.25^*^** | 1 |  |  |  |  |  |  |  |  |  |
| 12. Point to End (FPP) ‡ | -0.14 | -0.19 | **-0.21^*^** | **-0.4^**^** | -0.12 | **-0.23^*^** | **0.22^*^** | **0.49^**^** | **0.33^**^** | **0.47^**^** | 0.18 | 1 |  |  |  |  |  |  |  |  |
| 13. Perspective Taking (score) | -0.14 | -0.03 | **-0.43^**^** | **-0.2^*^** | -0.08 | -0.1 | 0.14 | **0.21^*^** | **0.45^**^** | 0.16 | **0.3^**^** | **0.29^**^** | 1 |  |  |  |  |  |  |  |
| 14. Corsi Span Forward (product) | **0.2^*^** | **0.27^**^** | 0.11 | **0.37^**^** | 0.1 | 0.08 | -0.1 | **-0.33^**^** | -0.17 | **-0.24^*^** | **-0.25^*^** | **-0.39^**^** | -0.15 | 1 |  |  |  |  |  |  |
| 15. Corsi Span Backward (product) | 0.12 | 0.06 | **0.24^*^** | 0.15 | 0.16 | 0 | **-0.2^*^** | -0.04 | -0.19 | -0.09 | -0.18 | **-0.2^*^** | **-0.32^**^** | **0.31^**^** | 1 |  |  |  |  |  |
| 16. Digit Span Forward (product) | -0.02 | 0.03 | 0.08 | 0.15 | -0.04 | 0.18 | 0.01 | -0.15 | 0.01 | -0.08 | -0.08 | -0.01 | -0.07 | 0.15 | 0.01 | 1 |  |  |  |  |
| 17. Digit Span Backward (product) | 0.19 | 0.08 | **0.21^*^** | **0.2^*^** | 0 | 0.15 | -0.07 | -0.09 | -0.11 | -0.01 | **-0.21^*^** | -0.11 | **-0.21^*^** | **0.21^*^** | 0.14 | **0.55^**^** | 1 |  |  |  |
| 18. Mental Rotation (accuracy) | 0.06 | 0.09 | **0.28^**^** | 0.11 | -0.02 | 0.13 | -0.16 | **-0.28^**^** | -0.19 | -0.17 | **-0.2^*^** | **-0.25^*^** | **-0.28^**^** | 0.19 | 0.13 | 0.08 | 0.16 | 1 |  |  |
| 19. Mental Rotation (reaction time) | 0 | 0.13 | 0.08 | **0.22^*^** | -0.03 | 0.1 | -0.07 | **-0.31^**^** | 0.04 | -0.15 | 0.01 | -0.18 | 0.1 | 0.05 | **-0.21^*^** | 0.06 | 0.04 | **0.5^**^** | 1 |  |
| 20. Mental Rotation (slope) | -0.04 | 0.03 | -0.03 | 0.03 | -0.09 | -0.02 | 0.04 | -0.15 | 0.06 | -0.11 | 0.07 | -0.04 | -0.05 | -0.1 | -0.15 | 0.11 | 0.07 | **0.43^**^** | **0.69^**^** | 1 |

_DMP = Dynamic Map perspective. FPP = First Person Perspective. † = Transformed using 10 for correlation analysis. ‡ = transformed using SQRT for correlation analysis._ **^*^** _Significance at p<.05_ **^**^** _Significance at p<0.01. Significant correlations printed in bold letters_
