# Supplementary material for: Spatial knowledge acquired from first-person and dynamic map perspectives
Source: Psychol Res. 2020 Aug 9;85(6):2137–50. doi: 10.1007/s00426-020-01389-y (PMC8357693; doi:10.1007/s00426-020-01389-y)
Supplement: Supplementary file 2 — Supplementary material 2 (PDF 354 kb) [file 426_2020_1389_MOESM2_ESM.pdf]

Supplemental table 2. Multiple regression diagnostics (First-person perspective)

Regression analysis	Normal distribution of residuals	homoscedasticity	Residual Independence	multicollinearity		Cook's Distance
	PP plot	scatterplot	Durbin-Watson	TOL	VIF	Maximum
**Route Sequence**	✓	✓	1.822	0.989	1.011	0.57
**Route Continuation**	✓	✓	1.881	0.974	1.027	0.96
**Distance Estimation**	N.S.	N.S.	N.S.	N.S.	N.S.	N.S.
**Location on Map**	✓	✓	1.807	0.985	1.015	0.1
**Point to Start**	✓	✓	1.968	0.953	1.049	0.157
**Point to End**	✓	✓	1.885	0.899	1.112	0.062
