# Supplementary material for: Spatial knowledge acquired from first-person and dynamic map perspectives
Source: Psychol Res. 2020 Aug 9;85(6):2137–50. doi: 10.1007/s00426-020-01389-y (PMC8357693; doi:10.1007/s00426-020-01389-y)
Supplement: Supplementary file 3 — Supplementary material 3 (PDF 354 kb) [file 426_2020_1389_MOESM3_ESM.pdf]

Supplemental table 3. Multiple regression diagnostics (dynamic map perspective)

**Regression analysis**	**Normal distribution of residuals**	**homoscedasticity**	**Residual Independence**	**multicollinearity**		**Cook's Distance**
	PP plot	scatterplot	Durbin-Watson	TOL	VIF	Maximum
**Route Sequence**	✓	✓	2.048	0.978	1.022	0.181
**Route Continuation**	✓	✓	1.1711	0.93	1.075	0.238
**Distance Estimation**	✓	✓	2.107	1.00	1.00	0.062
**Location on Map**	✓	✓	2.181	1.00	1.00	0.89
**Point to Start**	✓	✓	2.069	1.00	1.00	0.163
**Point to End**	✓	✓	2.152	0.974	1.027	0.088
